# COVID-19 impact on blood donor characteristics and seroprevalence of transfusion-transmitted infections in southern Thailand between 2018 and 2022

**DOI:** 10.1038/s41598-024-57584-z

**Published:** 2024-04-04

**Authors:** Suparat Burananayok, Wilaiwan Nachatri, Pimpilalai Choothanorm, Kantarat Kusolthammarat, Kanoot Jaruthamsophon, Chaninporn Yodsawad, Praopim Limsakul, Krit Charupanit

**Affiliations:** 1 Blood Bank and Transfusion Medicine Unit, Department of Pathology, Faculty of Medicine, Prince of Songkla University, Songkhla, Thailand; 2 Division of Physical Science, Faculty of Science, Prince of Songkla University, Songkhla, Thailand; 3 Department of Biomedical Sciences and Biomedical Engineering, Faculty of Medicine, Prince of Songkla University, Songkhla, Thailand; 4 Human Genetic Unit, Department of Pathology, Faculty of Medicine, Prince of Songkla University, Songkhla, Thailand; 5 Center of Excellence for Trace Analysis and Biosensor (TAB-CoE), Faculty of Science, Prince of Songkla University, Songkhla, Thailand

**Keywords:** HIV, HCV, HBV, Syphilis, Blood donation, Transfusion, Haematological diseases, Infectious diseases, Infection

## Abstract

Blood safety is a critical aspect of healthcare systems worldwide involving rigorous screening, testing, and processing protocols to minimize the risk of transfusion-transmitted infections (TTIs). The present study offers a comprehensive assessment of the prevalence of hepatitis B virus (HBV), hepatitis C virus (HCV), human immunodeficiency virus (HIV), and syphilis among blood donors in southern Thailand. It explores the consequences of the COVID-19 pandemic on the blood transfusion service, donor characteristics, and the prevalence of TTIs. A retrospective analysis of 65,511 blood donors between 2018 and 2022 was conducted at Songklanagarind Hospital, Thailand. The socio-demographic characteristics of the donors were examined using the Chi-square test to assess the relationship between TTIs serological positivity and donor characteristics. The donors were divided into pre-COVID-19 (2018–2019) and during COVID-19 (2020–2022) groups to evaluate the impacts of COVID-19. The study found that HBV had the highest overall prevalence at 243 per hundred thousand (pht), followed by syphilis (118 pht), HCV (32 pht), and HIV (31 pht) over a five-year period of study. After COVID-19, the prevalence of HBV decreased by 21.8%; HCV decreased by 2.1%; HIV increased by 36.4%; and syphilis increased by 9.2%. The socio-demographic characteristics and TTIs prevalence were significantly altered over time. This study provides insights into blood donor characteristics and TTIs prevalence in southern Thailand, highlighting the understanding of the impact of COVID-19 on the spread of TTIs.

## Introduction

Blood transfusion is a globally utilized medical intervention that has proven instrumental in saving millions of lives annually. However, despite its benefits, transfused patients are at risk of acquiring transfusion-transmissible infections (TTIs), such as human immunodeficiency virus (HIV), hepatitis B and C viruses (HBV and HCV), and *treponema pallidum*, the causative agent of syphilis. While TTIs are considered rare and infrequently reported in many countries, extensive screenings of collected blood are recommended by the World Health Organization^1^.

The burden of HIV, viral hepatitis, and sexually transmitted infections is substantial, accounting for over two million deaths annually, which represents 4% of all global deaths^2^. Each day, one million people become newly infected, contributing to the ongoing public health challenge^3^. The situation is particularly severe in the African region, where nearly a million people acquired HIV and half a million died in 2020^3^. However, there is a lack of recent large-scale studies on HIV prevalence among blood donors in Thailand.

HBV and HCV infections also pose significant risks, causing acute and chronic liver diseases, cirrhosis, and hepatocellular carcinoma among TTIs. In 2019, an estimated 296 and 58 million individuals were living with chronic HBV and HCV infections worldwide, respectively^4^. Approximately 1.5 million people acquire HBV and HCV infections each year^4^. In Thailand, around three million people, equivalent to 5.1% of the population, live with chronic HBV infections^5,6^. A 2016 study estimated that 759,000 individuals in Thailand are currently anti-HCV-positive, with 357,000 individuals having viremic HCV infection^7^.

Syphilis, a significant global health concern, is a sexually transmitted infection. In 2012, approximately 17.7 million individuals aged 15–49 had syphilis, with 5.6 million new cases reported annually^8,9^. In Thailand, syphilis has been recognized as a major sexually transmitted infection, as evidenced by its increased prevalence and co-infection with HIV^10–12^.

Globally, the highly contagious coronavirus 2019 (COVID-19), which is caused by the SARS-CoV-2 virus, has caused significant disruptions to daily life. With restrictions on population movements, social distancing measures, and disruptions in healthcare services, the COVID-19 pandemic has adversely affected blood transfusion services, leading to critical shortages in blood supply^13–15^. This study aims to evaluate the impact of COVID-19 on the socio-demographic characteristics of blood donors and the seroprevalence of TTIs, namely HIV, HBV, HCV, and syphilis, in southern Thailand. This study aims to provide insights into the spreading pattern of TTIs and the changes in donor characteristics. The goal is to enhance safety and ensure an adequate blood supply by strengthening screening protocols and implementing adaptive measures, such as diversifying the donor pool. These insights will inform public health interventions in response to the unexpected pandemic. Given that COVID-19 has not only directly impacted people's daily lives but also has broader implications, particularly for public healthcare, the results underscore the importance of understanding and adapting our systems to accommodate the ‘new normal' and prepare for an uncertain future.

## Results

During the period spanning from January 1st, 2018, to December 31st, 2022, information was gathered from a total of 65,511 blood donors (with an annual average of 13,102 ± 1511 donors) (Table 1). The overall socio-demographic profile of the blood donors was presented in Table 2. The gender distribution of the donors was almost equal, with males accounting for 47.2% of the total. The age range of the donors varied from 17 to 65 years old, with a mean age of 33.2 ± 11.1 years. Notably, the Blood Bank and Transfusion Medicine Unit, situated within Prince of Songkla University, primarily attracted university students as participants (25.1%), followed by government officers and state employees (20.4%).

**Table 1:** Number of donors who donated blood to Songklanagarind Hospital between 2018 and 2022.

Year	No. donor (N)	Percentage (%)
2018	13,893	21.2
2019	13,849	21.1
2020	13,887	21.2
2021	10,080	15.4
2022	13,802	21.1
Total	65,511	100

**Table 2:** Socio-demographic characteristics of blood donors and TTIs seroprevalence between 2018 and 2022 at Songklanagarind Hospital.

Donor characteristic	Number of donors		HBV prevalence		HCV prevalence		HIV prevalence		Syphilis prevalence	
	(N)	(%)	(N)	(pht)	(N)	(pht)	(N)	(pht)	(N)	(pht)
Gender										
Male	30,937	47.2	101	326	13	42	13	42	48	155
Female	34,574	52.8	58	168	8	23	7	20	29	84
χ^2^ (*p *value)			17.0 (< 0.001)		1.8 (0.178)		2.5 (0.111)		7.1 (0.008)	
Age										
< 21	8795	13.4	3	34	2	23	1	11	11	125
21–30	22,165	33.8	23	104	4	18	9	41	27	122
31–40	15,877	24.2	40	252	5	31	8	50	13	82
41–50	12,518	19.1	50	399	5	40	2	16	15	120
> 50	6156	9.4	43	699	5	81	0	0	11	179
χ^2^ (*p *value)			99.1 (< 0.001)		6.5 (0.166)		6.6 (0.159)		3.8 (0.439)	
Occupation										
Gov/state employee	13,356	20.4	41	631	2	17	3	25	12	215
Company employee	7930	12.1	16	202	3	38	3	38	6	76
Business owner	6689	10.2	26	389	3	45	0	0	15	224
Freelance	10,011	15.3	49	489	8	80	3	30	18	180
Farmer	1572	2.4	4	254	2	127	1	64	2	127
High school student	3100	4.7	0	0	0	0	0	0	2	65
University student	16,456	25.1	10	61	3	18	6	36	15	91
Cleric/Monk	270	0.4	2	741	0	0	0	0	0	0
Other	6127	9.4	11	377	0	0	4	159	7	218
χ^2^ (*p *value)			67.7 (< 0.001)		17.3 (0.028)		6.7 (0.572)		13.9 (0.084)	
Donor experience										
First-time donor	10,653	16.3	62	582	15	141	9	84	33	310
Repeat donor	54,858	83.7	97	177	6	11	11	20	44	80
χ^2^ (*p *value)			60.5 (< 0.001)		47.0 (< 0.001)		12.1 (< 0.001)		40.0 (< 0.001)	
Donation location										
At hospital	52,459	80.1	124	236	17	32	14	27	51	97
Mobile unit	13,052	19.9	35	268	4	31	6	46	26	199
χ^2^ (*p *value)			0.4 (0.509)		0.0 (0.92)		1.3 (0.259)		9.3 (0.002)	
Total	65,511	100	159	243	21	32	20	31	77	118

Regarding the blood type distribution in our study, the proportions were as follows: 38.9% for type O, 24.1% for type A, 29.5% for type B, and 7.5% for type AB (Supplementary S1). Among the donors, the majority were repeat donors, constituting approximately five times the number of new donors. Furthermore, approximately one-fifth of the blood donations (19.9%) were obtained through mobile units, while the remaining donations were received from individuals who donated blood at the Blood Bank and Transfusion Medicine Unit (80.1%). The socio-demographic characteristics of blood donors who contributed to Songklanagarind Hospital from January 2018 to December 2022 on a yearly basis were displayed in Supplementary S1.

Overall, the per-donation rates for HBV-positive donations throughout the entire study were 243 pht, followed by 118 pht for syphilis, 32 pht for HCV, and 31 pht for HIV. We observed that the prevalence of all TTIs in males was approximately double that of females. There was a significant relationship between HBV prevalence and age (χ^2^ = 99.1, *p* < 0.001), whereas no significant relationship was found for HCV, HIV, and syphilis (*p* > 0.05). The prevalence of HBV varied by occupation (χ^2^ = 67.7, *p* < 0.001), with clerics (741 pht) and government/state employees (631 pht) having the highest HBV prevalence. A significant difference in prevalence was also observed for HCV (χ^2^ = 17.3, *p* = 0.028). It was noted that the "other" category of occupations (included housewives, unemployed individuals, those with unidentified or no recorded occupations, and individuals with various careers with a small number). This category had the highest rate of HIV infection (159 pht). We found that the prevalence of TTIs among first-time donors was 3.3, 12.9, 4.2, and 3.9 times higher than that among repeat donors for HBV, HCV, HIV, and syphilis, respectively. A chi-square test indicated no significant association between the prevalence of HBV, HCV, and HIV and the location of donation. However, syphilis was found to be significantly more frequent among donors at mobile units than at the hospital (χ^2^ = 9.3, *p* = 0.002) (Table 2). Note that the characteristics were related in some categories e.g. students were mostly under 23 years old representing the majority of the younger donors and had lower TTIs prevalence. Additionally, supplementary information on prevalence by blood types can be found in Supplementary S2.

### Hepatitis B virus

Among the four TTIs studied, HBV had the highest prevalence. There was no significant relationship between HBV prevalence and the year of blood donation (χ^2^ = 6.7, *p* = 0.150). Overall, the prevalence of HBV in males was double that in females (χ^2^ = 17.0, *p* < 0.001). HBV was less likely to be found (84 pht) in younger donors (age < 30 years old) compared to 385 pht of the donor aged > 30 years old. The relationship between HBV prevalence and age was significant for each individual year during the study. HBV prevalence was significantly associated with occupation from 2018 to 2021. The top three occupations with the highest prevalence were clerics (741 pht), government/state employees (631 pht), and freelancers (489 pht), respectively. Surprisingly, there was a statistically significant difference in HBV prevalence among blood types (χ^2^ = 27.3, *p* < 0.001) (Supplementary S2). Similarly, there was a significant relationship between the donor's experience and HBV prevalence (χ^2^ = 60.5, *p* < 0.001). However, no significant association was found between prevalence and the location of donation in HBV (Table 3).

**Table 3:** Positivity rate of HBV by socio-demographic characteristics of the donors from 2018 to 2022.

Donor characteristic	HBV prevalence											
	2018		2019		2020		2021		2022		Five years	
	(N)	(pht)	(N)	(pht)	(N)	(pht)	(N)	(pht)	(N)	(pht)	(N)	(pht)
Gender												
Male	23	360	24	376	22	364	21	446	11	148	101	326
Female	13	173	17	228	12	153	6	112	10	157	58	168
χ^2^ (*p *value)	4.6 (0.031)		2.6 (0.109)		6.2 (0.013)		10.5 (0.001)		0.015 (0.9)		17.0 (< 0.001)	
Age												
< 20	0	0	2	98	1	46	0	0	0	0	3	34
21–30	3	59	7	147	8	169	4	124	1	23	23	104
31–40	10	296	11	333	9	278	7	282	3	86	40	252
41–50	13	538	9	353	10	396	11	507	7	244	50	399
> 51	10	953	12	997	5	498	5	459	10	622	41	699
χ^2^ (*p *value)	40.0 (< 0.001)		26.7 (< 0.001)		10.3 (0.035)		11.7 (0.020)		33.0 (< 0.001)		99.1 (< 0.001)	
Occupation												
Gov/State employee	6	784	12	802	7	593	9	736	7	229	41	631
Company employee	8	466	1	59	2	133	3	256	2	109	16	202
Business owner	5	338	6	418	7	515	3	289	5	363	26	389
Freelance	12	597	13	644	10	495	10	622	4	170	49	489
Farmer	0	0	1	307	1	289	1	376	1	303	4	254
High school student	0	0	0	0	0	0	0	0	0	0	0	0
University student	1	25	5	134	4	97	0	0	0	0	10	61
Cleric/Monk	1	1515	0	0	1	1538	0	0	0	0	2	741
Other	3	679	3	659	2	188	1	121	2	181	11	377
χ^2^ (*p *value)	27.1 (< 0.001)		20.7 (0.008)		20.1 (0.010)		16.5 (0.036)		10.4 (0.236)		67.7 (< 0.001)	
Donor experience												
First-time donor	16	716	15	687	12	496	8	567	11	458	62	582
Repeat donor	20	172	26	223	22	192	19	219	10	88	97	177
χ^2^ (*p* value)	21.5 (< 0.001)		13.4 (< 0.001)		7.6 (0.006)		5.5 (0.019)		17.9 (< 0.001)		60.5 (< 0.001)	
Donation location												
At hospital	29	256	32	295	28	253	20	242	15	137	124	236
Mobile unit	7	273	9	299	6	212	7	383	6	212	35	268
χ^2^ (*p *value)	0.0 (0.875)		0.0 (0.970)		0.2 (0.697)		1.1 (0.292)		0.8 (0.361)		0.4 (0.509)	
Total	36	259	41	296	34	245	27	268	21	152	159	243

### Hepatitis C virus

The prevalence of HCV was 7.5 times lower than that of HBV (32 pht and 243 pht, respectively). Over the five-year period, only 21 positive cases were found in the study, the second lowest positive cases, so there was no significant association between HCV prevalence and the socio-demographic characteristics collected, except for the donor's age in 2018, the donor's occupation, and the donor's experience. In addition, first-time donors tended to have a higher HCV prevalence (141 pht), which was 12.9 times higher than that among repeat donors (χ^2^ = 47.0, *p* < 0.001) (Table 4).

**Table 4:** HCV seroprevalence by socio-demographic characteristics of the blood donors from 2018 to 2022.

Donor characteristic	HCV prevalence											
	2018		2019		2020		2021		2022		Five years	
	(N)	(pht)	(N)	(pht)	(N)	(pht)	(N)	(pht)	(N)	(pht)	(N)	(pht)
Gender												
Male	3	47	3	47	4	66	2	43	1	13	13	42
Female	2	27	1	13	3	38	0	0	2	31	8	23
χ^2^ (*p* value)	0.4 (0.531)		1.3 (0.246)		0.5 (0.468)		2.3 (0.131)		0.5 (0.479)		1.8 (0.178)	
Age												
< 20	0	0	1	49	1	46	0	0	0	0	2	23
21–30	1	20	0	0	1	21	1	31	1	23	4	18
31–40	0	0	1	30	2	62	0	0	2	58	5	31
41–50	2	83	1	39	1	40	1	46	0	0	5	40
> 51	2	191	1	83	2	166	0	0	0	0	5	81
χ^2^ (*p* value)	10.7 (0.030)		3.0 (0.561)		4.2 (0.385)		1.9 (0.756)		3.3 (0.502)		6.5 (0.166)	
Occupation												
Gov/state employee	0	0	0	0	0	0	0	0	2	65	2	17
Company employee	1	58	0	0	2	133	0	0	0	0	3	38
Business owner	0	0	1	70	1	74	0	0	1	73	3	45
Freelance	2	99	2	99	3	149	1	62	0	0	8	80
Farmer	1	329	0	0	0	0	1	376	0	0	2	127
High school student	0	0	0	0	0	0	0	0	0	0	0	0
University student	1	25	1	27	1	24	0	0	0	0	3	18
Cleric/Monk	0	0	0	0	0	0	0	0	0	0	0	0
Other	0	0	0	0	0	0	0	0	0	0	0	0
χ^2^ (*p *value)	12.0 (0.151)		6.2 (0.624)		9.0 (0.339)		20.1 (0.010)		5.8 (0.667)		17.3 (0.027)	
Donor experience												
First-time donor	3	134	4	183	4	165	2	142	2	83	15	141
Repeat donor	2	17	0	0	3	26	0	0	1	9	6	11
χ^2^ (*p *value)	7.1 (0.008)		21.4 (< 0.001)		7.7 (0.006)		12.3 (< 0.001)		5.1 (0.024)		47.0 (< 0.001)	
Donation location												
At hospital	3	26	4	37	5	45	2	24	3	27	17	32
Mobile unit	2	78	0	0	2	71	0	0	0	0	4	31
χ^2^ (*p *value)	1.5 (0.213)		1.1 (0.292)		0.3 (0.588)		0.4 (0.506)		0.8 (0.379)		0.0 (0.920)	
Total	5	36	4	29	7	50	2	20	3	22	21	32

### Human immunodeficiency virus

HIV had the lowest number of positive cases (20 cases) among four TTIs. Therefore, we found statistically significant relationships only between the blood type and the donor's experience with HIV prevalence, but these differences were not consistent over the study period. HIV prevalence significantly differed by blood type only in 2020 (χ^2^ = 12.5, *p* = 0.006) and 2022 (χ^2^ = 13.4, *p* = 0.004) (Supplementary S2). The relationship between HIV prevalence and the donor's experience was significant only in 2018 (χ^2^ = 11.4, *p* < 0.001) and 2022 (χ^2^ = 9.2, *p* = 0.002). In terms of age distribution, the highest age among HIV-positive cases was 46 years old, while the age ranges with the highest HIV prevalence were 21–30 (41 pht) and 31–40 years old (50 pht). Additionally, the occupation with the highest HIV prevalence was housewife/unemployed, categorized under the "other" section (Table 5).

**Table 5:** HIV seroprevalence by socio-demographic characteristics of the blood donors from 2018 to 2022.

Donor characteristic	HIV prevalence											
	2018		2019		2020		2021		2022		Five years	
	(N)	(pht)	(N)	(pht)	(N)	(pht)	(N)	(pht)	(N)	(pht)	(N)	(pht)
Gender												
Male	4	63	0	0	2	33	3	64	4	54	13	42
Female	2	27	1	13	2	26	2	37	0	0	7	20
χ^2^ (*p *value)	1.0 (0.310)		0.9 (0.355)		0.1 (0.794)		0.4 (0.550)		3.4 (0.063)		2.5 (0.111)	
Age												
< 20	0	0	1	49	0	0	0	0	0	0	1	11
21–30	1	20	0	0	2	42	4	124	2	46	9	41
31–40	4	119	0	0	1	31	1	40	2	58	8	50
41–50	1	41	0	0	1	40	0	0	0	0	2	16
> 51	0	0	0	0	0	0	0	0	0	0	0	0
χ^2^ (*p *value)	6.4 (0.171)		5.8 (0.216)		1.4 (0.848)		5.8 (0.212)		3.1 (0.538)		6.6 (0.159)	
Occupation												
Gov/state employee	0	0	0	0	0	0	2	92	1	33	3	25
Company employee	1	58	0	0	1	66	0	0	1	54	3	38
Business owner	0	0	0	0	0	0	0	0	0	0	0	0
Freelance	1	50	0	0	0	0	2	124	0	0	3	30
Farmer	1	329	0	0	0	0	0	0	0	0	1	64
High school student	0	0	0	0	0	0	0	0	0	0	0	0
University student	1	25	1	27	2	48	1	51	1	37	6	36
Cleric/Monk	0	0	0	0	0	0	0	0	0	0	0	0
Other	2	587	0	0	1	94	0	0	1	91	4	159
χ^2^ (*p *value)	11.9 (0.157)		2.7 (0.951)		4.4 (0.817)		4.3 (0.825)		2.8 (0.946)		6.7 (0.572)	
Donor experience												
First-time donor	4	179	0	0	2	83	0	0	3	125	9	84
Repeat donor	2	17	1	9	2	17	5	58	1	9	11	20
χ^2^ (*p *value)	11.4 (< 0.001)		0.2 (0.665)		3.0 (0.086)		0.8 (0.367)		9.2 (0.002)		12.1 (< 0.001)	
Donation location												
At hospital	3	26	0	0	3	27	5	61	3	27	14	27
Mobile unit	3	117	1	33	1	35	0	0	1	35	6	46
χ^2^ (*p *value)	4.0 (0.046)		3.6 (0.058)		0.1 (0.817)		1.1 (0.293)		0.0 (0.825)		1.3 (0.259)	
Total	6	43	1	7	4	29	5	50	4	29	20	31

### Syphilis

Syphilis had the second-highest prevalence in the study (118 pht). Although a chi-square test of independence showed that the overall syphilis prevalence was significantly associated with gender (χ^2^ = 7.1, *p* = 0.008) with the prevalence among male donors consistently higher than that among females in every individual year, there was no significant relationship between the two variables in any single year during our study. In terms of age, the results showed that syphilis prevalence was statistically associated with age only during 2020–2021. First-time donors were more likely to test positive for syphilis than repeat donors in 2019–2022 (*p* < 0.05). There was a significant relationship between syphilis prevalence and donation location in 2018. Surprisingly, syphilis and HIV shared the same trend, with prevalence from donations at mobile units being nearly double that at the hospital, which was much higher compared to HBV and HCV (Table 6).

**Table 6:** Syphilis seroprevalence by socio-demographic characteristics of the blood donors from 2018 to 2022.

Donor characteristic	Syphilis prevalence											
	2018		2019		2020		2021		2022		Five years	
	(N)	(pht)	(N)	(pht)	(N)	(pht)	(N)	(pht)	(N)	(pht)	(N)	(pht)
Gender												
Male	6	94	12	188	10	165	11	234	9	121	48	155
Female	5	67	8	107	7	89	5	93	4	63	29	84
χ^2^ (*p *value)	0.3 (0.570)		1.6 (0.211)		1.6 (0.203)		3.1 (0.076)		1.3 (0.261)		7.1 (0.008)	
Age												
< 20	0	0	5	245	1	46	3	268	2	136	11	125
21–30	3	59	7	147	3	63	10	311	4	91	27	122
31–40	3	89	2	61	5	154	1	40	2	58	13	82
41–50	3	124	5	196	3	119	1	46	3	105	15	120
> 51	2	191	1	83	5	415	1	92	2	124	11	179
χ^2^ (*p *value)	4.1 (0.388)		3.8 (0.429)		11.1 (0.025)		9.8 (0.045)		1.0 (0.915)		3.8 (0.439)	
Occupation												
Gov/state employee	2	99	2	88	2	375	3	137	3	398	12	215
Company employee	1	58	2	118	2	133	1	85	0	0	6	76
Business owner	2	135	4	279	4	294	3	289	2	145	15	224
Freelance	3	149	5	248	7	347	2	124	1	42	18	180
Farmer	0	0	0	0	0	0	1	376	1	303	2	127
High school student	1	140	0	0	0	0	1	178	0	0	2	65
University student	1	25	7	188	1	24	3	154	3	111	15	91
Cleric/Monk	0	0	0	0	0	0	0	0	0	0	0	0
Other	1	92	0	0	1	94	2	243	3	644	7	218
χ^2^ (*p *value)	4.0 (0.857)		8.1 (0.423)		16.7 (0.033)		2.8 (0.945)		7.4 (0.495)		13.9 (0.084)	
Donor experience												
First-time donor	3	134	8	367	7	289	10	708	5	208	33	310
Repeat donor	8	69	12	103	10	87	6	69	8	70	44	80
χ^2^ (*p* value)	1.0 (0.312)		8.9 (0.003)		6.7 (0.010)		31.3 (< 0.001)		4.0 (0.045)		40.0 (< 0.001)	
Donation location												
At hospital	5	44	15	138	11	99	12	145	8	73	51	97
Mobile unit	6	234	5	166	6	212	4	219	5	176	26	199
χ^2^ (*p* value)	9.6 (0.002)		0.1 (0.721)		2.4 (0.125)		0.5 (0.475)		2.6 (0.109)		9.3 (0.002)	
Total	11	79	20	144	17	122	16	159	13	94	77	118

### Transfusion-transmitted infections prevalence and household Income

There are evidences linking TTIs prevalence to income^16–24^. Therefore, we aimed to determine the relationship between TTIs prevalence and household income. The participant donors in our study were from various parts of Thailand, all 77 provinces of Thailand. However, for the purpose of this analysis, we focused on donors from the southern region of Thailand, 14 provinces, which accounted for 63,222 out of 65,511 donors (96.5%). We selected this region because it had a sufficient number of positive cases. Thus, the study included a total of 62,756 donors (95.8%) from 12 non-zero TTIs positive provinces (Supplementary S3).

The household income used in our study was estimated based on the average value from the Thailand household income national reports between 2019 and 2021^25^. The currency conversion rate used was 34.5 Thai Baht = $1 USD. To assess the linear relationship between the total TTIs prevalence and household income, we computed a Pearson correlation coefficient. It is important to note that we used the total prevalence, which represents the combined prevalence of all four TTIs since there were insufficient positive cases to draw conclusions regarding the relationship between HCV and HIV. Therefore, this analysis heavily relied on HBV positives (56.6%) and syphilis (27.3%).

**Figure 1:**
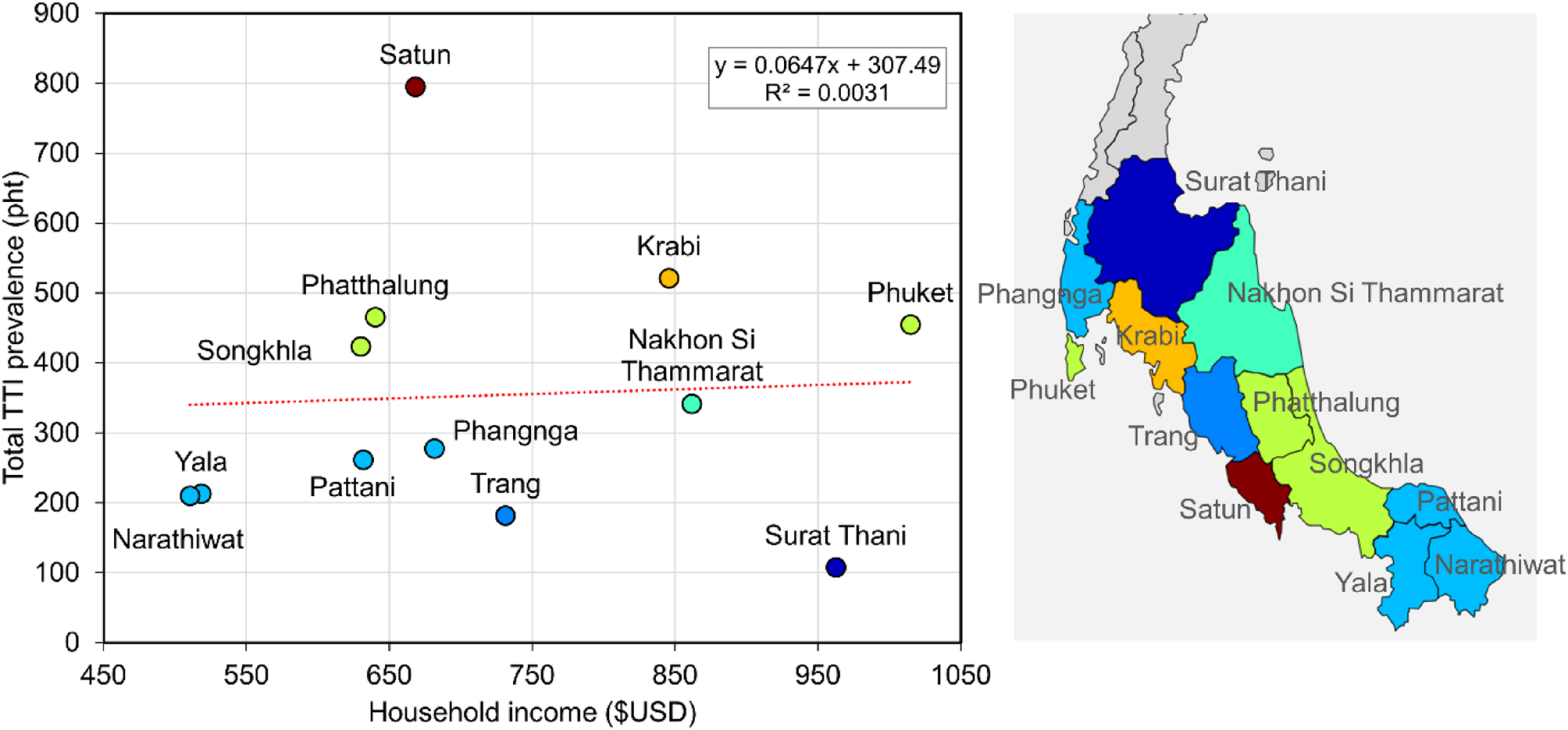
Pooled TTIs prevalence of 12 non-zero TTIs positive provinces of southern Thailand (right: geographical map of southern Thailand) and the average monthly income per household between 2019 and 2021.

The results showed no correlation between the two variables, with a correlation coefficient of r(10) = 0.06 and a *p* value of 0.861 (Fig. 1).

### COVID-19 impact on prevalence of transfusion-transmitted infections

The COVID-19 pandemic, which began in late 2019, had a profound global impact, leading to significant disruptions and changes worldwide. In our study, we examined the effect of COVID-19 on the prevalence of TTIs among blood donors in Thailand. The dataset was divided into two periods based on the year of donation: pre-COVID-19 (2018–2019) and during COVID-19 (2020–2022). The number of donors were 27,742 and 37,769, respectively.

**Figure 2:**
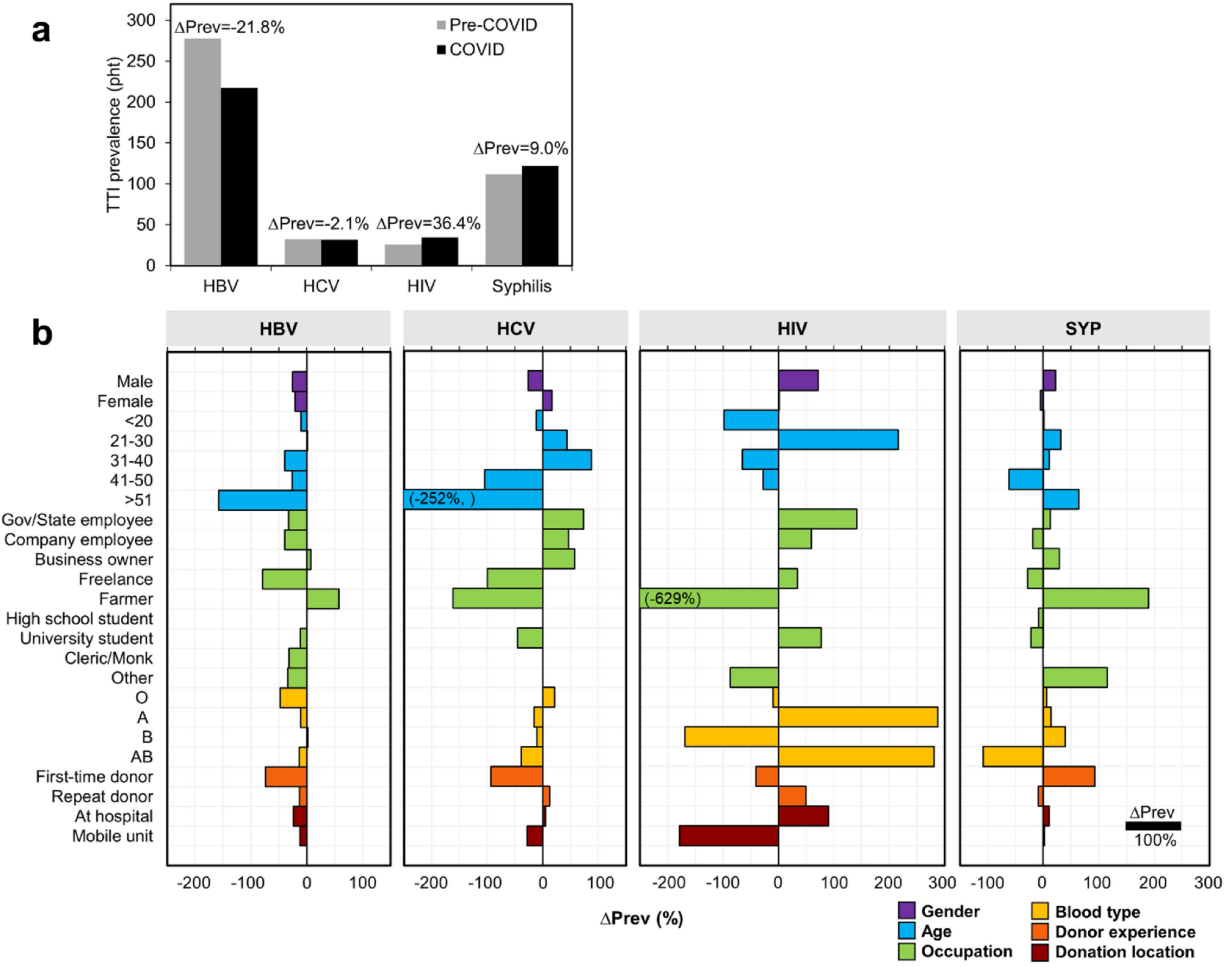
(**a**) The prevalence of HBV, HCV, HIV, and syphilis comparing between pre-COVID (2018–2019) and during pandemic (2020–2022), and their prevalence difference (∆Prev). (**b**) The ∆Prev of TTIs comparing between pre-COVID pandemic (2018–2019) and during pandemic (2020–2022) by socio-demographic characteristics of the blood donors. Note that the number in parentheses on the bar represents the actual value that exceeds the axis limit.

Our findings revealed slight alterations in the prevalence of HCV and syphilis, with a decrease of 2.1% and an increase of 9.2%, respectively (Fig. 2a). However, the prevalence of HBV was significantly reduced by 21.8% (from 278 to 217 pht), while the HIV prevalence showed an increase of 36.4% comparing the pre-COVID-19 period (25 pht) to the COVID-19 period (34 pht). To illustrate the changes between the pre-COVID-19 and during COVID-19 periods, we presented the difference in TTIs prevalence normalized by the overall prevalence of each respective TTIs during the pre-COVID-19 period in percentage form (∆Prev) (Fig. 2).

Due to the substantial reduction in HBV prevalence during the COVID-19 period, the HBV prevalence for each individual socio-demographic characteristic of blood donors mostly decreased accordingly. Among HBV cases, the highest decrease in prevalence (158%) was observed in elder donors (> 51 years old), while the highest increase (58%) occurred among donors who were farmers. Additionally, the prevalence of HBV among first-time donors decreased by 74% after the onset of COVID-19.

Given the limited number of positive cases of HCV, there was not a significant change in the actual HCV prevalence over time. However, in terms of percentage change, there was a noticeable decrease in HCV prevalence among donors aged over 40 years, freelance and farmer occupations, and new donors. Conversely, HCV prevalence increased by 44% to 88% among adults aged 21–30 and 31–40 years old. Moreover, an increase in HCV prevalence was observed among government/state employees, company employees, and business owners.

The prevalence of HIV exhibited the most fluctuation among the studied TTIs. We found that the HIV prevalence among male donors increased by 72%, while it remained unchanged among females. The only age range that experienced an increase in HIV prevalence was 21–30 years old (217%). Furthermore, the change in HIV prevalence varied across occupations. Although the prevalence of HIV among new donors decreased by 41%, there was a 50% increase among repeat donors. The rate of HIV-positive cases found at mobile units decreased by 179%, in contrast to a 90% increase at the Blood Bank and Transfusion Medicine unit.

Regarding syphilis, the prevalence increased by 22% among males (with a 5% decrease among females). Syphilis was more frequently found in the younger generation (17–40 years old) and late adults (> 51 years old) during the COVID-19 period. Conversely, there was a significant decrease in syphilis prevalence among the group of 41–50 years old. Noticeable increases in syphilis prevalence were observed among farmers and occupations categorized as "other." The prevalence of syphilis among first-time donors nearly doubled, while a 9% decrease was observed among repeat donors. Additionally, the prevalence of syphilis slightly increased at both hospitals and mobile units (11% and 3% increases, respectively).

These results demonstrated that COVID-19 not only directly affects people's daily lives but also has an impact that extends to other aspects, such as public healthcare. Therefore, it is necessary for us to understand and adapt our systems in response to the new normal.

## Discussion

Addressing blood safety and availability poses significant challenges considering recent global shifts. This study focuses on the prevalence of TTIs, including HIV, HBV, HCV, and syphilis, across the southern region of Thailand over a five-year period. It examines both pre-COVID-19 and COVID-19 eras, explaining alterations in blood donor characteristics. The prevalence of TTIs increased for HIV (from 25 to 34 pht, 36.4%) and syphilis (from 112 to 122 pht, 9.2%), while decreasing for HBV (from 278 to 217 pht, − 21.8%) and HCV (32 to 32 pht, − 2.1%) following the pandemic onset. This research offers crucial insights into the consequences of a widespread pandemic and the influence of regulatory measures on people's social behavior through blood donation by tracking trends in TTI prevalence and donor characteristic patterns.

The number of blood donors at the Blood Bank and Transfusion Medicine Unit of Songklanagarind Hospital remained almost consistent over the five-year study period, even during the COVID-19 pandemic. There were approximately 13,000 donors per year, except for 2021, which saw a decrease to 10,080 (a 27% reduction). This decline contrasted with the average decrease in blood donations around the world, which was 38% during the same period^13–15,26^. The reduction in donation was a direct consequence of the COVID-19 situation in Thailand, which peaked from late 2020 through 2021. At the beginning of 2021, the first batch of COVID-19 vaccines was distributed, raising awareness of COVID-19 and resulting in social distancing and home quarantine throughout the year. However, the number of donors rebounded to normal levels in 2022. Although the occupational distribution of donors remained mostly consistent during the five-year study, the number of university student donors reduced by approximately half in 2021 due to the closure of university classes caused by COVID-19. To compensate, efforts were made to recruit more local donors and government/state employees. As Prince of Songkla University and Songklanagarind Hospital are public organizations, staff and employees who continued to work offline were actively encouraged to donate blood via social media campaigns and local advertisements, including posters and short advertisements displayed on monitors within the hospital premises.

Our study found that the prevalence of HBV was 243 pht (0.24%), which was higher than that reported in the US in 2015–2016^27^ and various countries in Europe and Japan^28,29^. However, it was significantly lower than reports from Africa and other countries^28–32^. The prevalence of HCV was 32 pht (0.03%), with an HBV/HCV ratio of 7.6 This contrasted with a previous report in 2022 that most countries around the world had an HBV/HCV ratio below one^33^. The HCV prevalence in our study was considered low compared to previous studies^30–37^, and it closely resembled the HCV prevalence in the US in the late 2010s^27^. Among the four TTIs, HIV had the lowest number of positive cases, with a prevalence of 31 pht (0.03%). The HIV prevalence in our study was considered low compared to recent reports and was similar to the prevalence in Japan, China, and South Korea^27,38–42^. Furthermore, our results showed that the prevalence of HIV was much lower than the global average (476 pht), Europe (254 pht), Asia (100 pht), Americas (391 pht), and Africa (1990 pht)^38^. In the case of syphilis, the prevalence was 118 pht (0.12%) in our study. Although this value was higher than that reported in Europe, the USA, and Australia, it was considerably lower than in Africa region^30,32,34,40,43–45^.

Although previous studies have suggested a link between TTIs prevalence and income, these studies mostly focused on national-level income^16–24^. In our study, we examined the TTIs prevalence and household income of donors in the South of Thailand, on the local economic scale. The results revealed no relationship between these variables. It is important to note that the population included in this analysis was heavily skewed, as the majority of donors were citizens of Songkhla province. Additionally, the household income used was based on the national report of provincial household income, which may not accurately represent the actual household income of individual blood donors. Therefore, the results may differ from previous studies.

It became evident that social interactions were reduced during the pandemic, which was expected to result in a drop in TTIs prevalence. However, we found that only the decrease in HBV prevalence was noticeable (21.8%). On the contrary, both HIV and syphilis prevalence increased during the pandemic, as they affected similar population groups^46,47^. However, the increase in prevalence was possibly due to false positive serological screening. Recent reports have shown that SARS-CoV-2 is linked to false positives in HIV screening because of its structural similarity^48–50^. Additionally, COVID-19 mRNA vaccines can cause false reactivity in some serologic laboratory tests for syphilis^51,52^. Therefore, follow-up studies of HIV and syphilis are necessary to understand the actual TTI seroprevalence situation during and after COVID-19. The impact of COVID-19 on HBV and HCV prevalence was clear in the decrease of prevalence among adult and elder donors (age over 40 years old). Moreover, we observed alterations in the distribution of positive cases among donor occupations after the pandemic, although the changes were inconsistent among the four TTIs. Surprisingly, the prevalence of first-time donors decreased in HBV, HCV, and HIV, while only syphilis showed a positive prevalence increase. Furthermore, except for HIV, there was no noticeable impact of COVID-19 on the relationship between TTIs prevalence and blood donation location.

Among the four TTIs, HBV has the highest prevalence in Thailand (243 pht). As in 1992, Thailand launched the Expanded Program on Immunization in which Hepatitis B Vaccine Injection is required at birth, the majority of the infected were donors aged over 30 years old, in agreement with previous reports^6,53^. Furthermore, we found that the HBV prevalence in males is double that of females, which might result from behavioral risk factors, such as tattooing, ear or body piercing, and sharing personal items, particularly among groups such as monks and government employees, including soldiers and police officers^54–57^. Therefore, public relations campaigns and initiatives are encouraged to the target population, especially those born before 1992, to undergo screening for hepatitis B virus. Utilizing media channels to continuously disseminate knowledge and educate on preventing and risks of HBV infection proactively. Furthermore, enhancing the precautionary measures of healthcare personnel in screening the medical history or risk behaviors of blood donors. This is coupled with providing information to instill awareness of the safety of blood that could adversely affect the health of patients receiving blood transfusions.

Despite including a large dataset of 65,511 blood donors in our study, the prevalence of TTIs, especially in HCV and HIV (21 and 20 cases, respectively), was low. Therefore, caution must be exercised when interpreting the results for HCV and HIV, as the prevalence fluctuated greatly between consecutive years due to the low number of positive cases. However, several consistent findings were observed in our study. For instance, although there were only 20 cases of positive HIV over the five-year period, six out of ten positive donors aged 17–30 years old were university students, resulting in one to two HIV-positive cases annually. Additionally, the substantial swing in HIV prevalence between the pre- and during-pandemic periods was caused by only one to two positive cases. Furthermore, 63,222 donors (96.5%) represented the population of the South of Thailand, indicating that our study may not fully represent the entire population of Thailand. In addition, as all four studied TTIs can be transmitted through sexual intercourse, and both COVID-19 infection and vaccination can cause false positives, information regarding sexual behavior, exposure to TTIs risk, and COVID-19 infection/vaccination history among donors are crucial factors. Unfortunately, these factors were not included as the records were limited; therefore, the changes in seroprevalence of TTIs must be interpreted with caution.

## Methods

In this population-based study, we combined individual-level data from the Songklanagarind Hospital digital database, Hospital Information System (HIS). The Human Research Ethics Committee, Faculty of Medicine, Prince of Songkla University, approved our study in 2022 (REC. 65.520-5-8) and waived the requirement for informed consent. All methods were performed in accordance with the relevant guidelines and regulations.

### Study population and data collection

This retrospective study was conducted at the Blood Bank and Transfusion Medicine Unit of Songklanagarind Hospital, located in southern Thailand, and focused on eligible blood donors from 2018 to 2022. The socio-demographic information of the participants was obtained from the HIS, the hospital's electronic database, which included variables such as gender, age, domicile, blood type, donation experience (first-time/repeat), recruiting site (at the hospital/mobile site), occupation, year of donation, and laboratory infection testing results (HIV, HBV, HCV, and syphilis). Additionally, the relationship between TTIs prevalence and provincial household income, as reported in the Thailand household income national reports between 2019 and 2021, was examined, considering evidence linking TTIs prevalence to income^16–24^. Participants were considered eligible for blood donation after undergoing medical screening in accordance with the guidelines established by the National Blood Centre, Thai Red Cross Society (Supplementary S5)^58^. A total of 65,511 blood donors were included in the study.

### Transfusion-transmitted infections screening

The screening process for TTIs, including HBV, HIV, HCV, and syphilis, was conducted by the Blood Bank and Transfusion Medicine Unit, Songklanagarind Hospital. To ensure the utmost safety of blood transfusions, two layers of screening were implemented to cross-verify the results. The first layer of safety consisted of serological tests using electrochemiluminescence immunoassay (ECLIA), a quantitative method for measuring antigens or antibodies based on the change in electrochemiluminescence, together with the rapid antigen tests (HIV, HBV, and HCV) and the rapid plasma reagin test (syphilis). The Nucleic Acid Amplification Technique (NAT), a molecular technique for screening, served as the second layer of safety.

Initially, three 6-ml samples of peripheral venous blood were collected from blood donors using ethylenediaminetetraacetic acid tubes for three laboratory screening tests. The first sample underwent ABO grouping and RhD typing. The second sample was subjected to serological screening for the presence of HIV antigen/antibody (HIV Ag/Ab), Hepatitis B surface antigen (HBsAg), antibodies to HCV (anti-HCV), and syphilis (treponemal antibody). The remaining sample was reserved for NAT screening. At the Blood Bank and Transfusion Medicine Unit, if either the serology or NAT results were positive, the sample was considered positive for that particular TTI.

The serological tests for HIV, HBV, HCV, and syphilis were conducted using the ECLIA technique on the Cobas e801 analyzer (Roche Diagnostics Corp., USA). Additionally, rapid antigen tests for HIV, HBV, and HCV (InTec Product Inc., China), as well as the rapid plasma reagin test for syphilis, were performed to crosscheck the diagnostic results. NAT, based on the amplification of targeted regions of viral ribonucleic acid or deoxyribonucleic acid, was employed as a cross-confirmation test due to its high sensitivity and specificity for viral nucleic acids, which can detect infections earlier than other screening methods, thereby narrowing the window period for HIV, HBV, and HCV infections. NAT was performed using the Cobas 6800 system (Roche Diagnostics Corp., USA).

### Statistical analysis

The collected data was subsequently subjected to a thorough cleansing process using an Excel spreadsheet (Microsoft Corp.). The statistical analysis was conducted using MATLAB 2018b (MathWorks Inc., USA) and R 4.2.1 (R Core Team, 2021). Socio-demographic variables with numerical values were presented in the format of mean ± standard deviation, while categorical variables such as gender, age range, donor occupation, blood type, donor experience, and recruiting site were summarized as proportions.

To assess the prevalence of TTIs, the number of seropositive samples per 100,000 donors (per hundred thousand; pht) was calculated. The relationship between categorical variables was examined through a chi-square (χ^2^) test of independence. Additionally, a Pearson correlation analysis was employed to determine the correlation between the prevalence of TTIs and household income. Statistical significance was considered when the *p* value was < 0.05.

## Conclusion

This study sheds light on the prevalence of TTIs in the southern region of Thailand over a five-year period, encompassing both pre-COVID-19 and during COVID-19 periods. Monitoring the prevalence trends of TTIs in blood donors serves as a valuable index for evaluating intervention strategies' effectiveness and understanding the impact of COVID-19 on TTIs and people's behavior. Our findings highlight variations in prevalence compared to other countries and regions. The influence of the pandemic on TTIs prevalence caused a 21.8% and 2.1% reduction in HBV and HCV, while HIV and syphilis increased by 36.4% and 9.0%, respectively. Furthermore, our study found no relationship between TTIs prevalence and household income at the local level. Further research and proactive surveillance efforts are necessary to enhance blood safety and availability in the face of ongoing global changes.

### Supplementary Information

Supplementary Information.

## Data Availability

The datasets used and/or analyzed during the current study available from the corresponding author (K.C.) on reasonable request.
