# Supplementary material for: COVID-19 impact on blood donor characteristics and seroprevalence of transfusion-transmitted infections in southern Thailand between 2018 and 2022
Source: Sci Rep. 2024 Apr 4;14:7920. doi: 10.1038/s41598-024-57584-z (PMC10995202; doi:10.1038/s41598-024-57584-z)
Supplement: Supplementary file 1 — Supplementary Information. [file 41598_2024_57584_MOESM1_ESM.pdf]

COVID-19 impact on characteristics and seroprevalence of transfusion-transmitted infections among blood donors in southern Thailand between 2018-2022

Suparat Burananayok[1], Wilaiwan Nachatri[1], Pimpilalai Choothanorm[1], Kantarat Kusolthammarat[1], Kanoot Jaruthamsophon[1], Chaninporn Yodsawad[1], Praopim Limsakul[2], Krit Charupanit[3,*]

[1]Blood Bank and Transfusion Medicine Unit, Department of Pathology, Faculty of Medicine, Prince of Songkla University.

[2]Division of Physical Science, Faculty of Science, Prince of Songkla University.

[3]Department of Biomedical Sciences and Biomedical Engineering, Faculty of Medicine, Prince of Songkla University

*Corresponding author; email: krit.ch@psu.ac.th

Supplementary S1

Socio-demographic characteristics of blood donors who contributed to Songklanagarind Hospital from January 2018 to December 2022 by year.

Donor Characteristic	2018		2019		2020		2021		2022		Five years	
	N	%	N	%	N	%	N	%	N	%	N	%
**Gender**												
Male	6,394	46.0	6,380	46.1	6,046	43.5	4,704	46.7	7,413	53.7	30,937	47.2
Female	7,499	54.0	7,469	53.9	7,841	56.5	5,376	53.3	6,389	46.3	34,574	52.8
**Age**												
17-20	1,986	14.3	2,041	14.7	2,179	15.7	1,120	11.1	1,469	10.6	8,795	13.4
21-30	5,067	36.5	4,756	34.3	4,739	34.1	3,220	31.9	4,383	31.8	22,165	33.8
31-40	3,374	24.3	3,301	23.8	3,242	23.3	2,482	24.6	3,478	25.2	15,877	24.2
41-50	2,417	17.4	2,547	18.4	2,523	18.2	2,168	21.5	2,863	20.7	12,518	19.1
51-65	1,049	7.6	1,204	8.7	1,204	8.7	1,090	10.8	1,609	11.7	6,156	9.4
**Occupation**												
Gov/State employee	2,370	17.1	2,580	18.6	2,595	18.7	2,453	24.3	3,358	24.3	13,356	20.4
Company employee	1,715	12.3	1,701	12.3	1,509	10.9	1,170	11.6	1,835	13.3	7,930	12.1
Business owner	1,480	10.7	1,434	10.4	1,360	9.8	1,039	10.3	1,376	10.0	6,689	10.2
Freelance	2,011	14.5	2,019	14.6	2,020	14.5	1,608	16.0	2,353	17.0	10,011	15.3
Farmer	304	2.2	326	2.4	346	2.5	266	2.6	330	2.4	1,572	2.4
High school student	713	5.1	729	5.3	601	4.3	562	5.6	495	3.6	3,100	4.7
University student	3,942	28.4	3,730	26.9	4,138	29.8	1,946	19.3	2,700	19.6	16,456	25.1
Cleric/Monk	66	0.5	61	0.4	65	0.5	41	0.4	37	0.3	270	0.4
Other	1,292	9.3	1,269	9.2	1,253	9.0	995	9.9	1,318	9.5	6,127	9.4
**Blood type**												
O	5,351	38.5	5,385	38.9	5,368	38.7	4,003	39.7	5,388	39.0	25,495	38.9
A	3,382	24.3	3,303	23.9	3,364	24.2	2,418	24.0	3,347	24.3	15,814	24.1
B	4,105	29.5	4,132	29.8	4,127	29.7	2,905	28.8	4,027	29.2	19,296	29.5
AB	1,055	7.6	1,029	7.4	1,028	7.4	754	7.5	1,040	7.5	4,906	7.5
**Donor experience**												
First-time donor	2,235	16.1	2,182	15.8	2,420	17.4	1,412	14.0	2,404	17.4	10,653	16.3
Repeat donor	11,658	83.9	11,667	84.2	11,467	82.6	8,668	86.0	11,398	82.6	54,858	83.7
**Donation location**												
At hospital	11,333	81.6	10,842	78.3	11,063	79.7	8,253	81.9	10,968	79.5	52,459	80.1
Mobile unit	2,560	18.4	3,007	21.7	2,824	20.3	1,827	18.1	2,834	20.5	13,052	19.9
**Total**	**13,893**	**100.0**	**13,849**	**100.0**	**13,887**	**100.0**	**10,080**	**100.0**	**13,802**	**100.0**	**65,511**	**100.0**

Supplementary S2

Blood types of the donors and prevalence of TTIs in a five-year study

Donor Characteristic	Sample		HBV prevalence		HCV prevalence		HIV prevalence		SYP prevalence	
	(N)	(%)	(N)	(pht)	(N)	(pht)	(N)	(pht)	(N)	(pht)
**Blood type**										
O	25,495	38.9	35	137	1	4	2	8	20	78
A	15,814	24.1	54	341	9	57	9	57	18	114
B	19,296	29.5	64	332	9	47	7	36	33	171
AB	4,906	7.5	6	122	2	41	2	41	6	122
χ^2 (*p*-value)			27.3(<0.001)		10.7(0.013)		8.3(0.040)		8.0(0.045)	
**Total**	**65,511**	**100**	**159**	**243**	**21**	**32**	**20**	**31**	**77**	**118**

Positivity rate of TTIs by blood type of the donors from 2018 to 2022 by year.

Donor Characteristic	2018		2019		2020		2021		2022		Five years	
	(N)	(pht)	(N)	(pht)	(N)	(pht)	(N)	(pht)	(N)	(pht)	(N)	(pht)
	**HBV prevalence**											
**Blood type**												
O	9	168	14	260	5	93	5	125	2	37	35	137
A	15	444	9	272	16	476	4	165	10	299	54	341
B	10	244	17	411	13	315	15	516	9	223	64	332
AB	2	190	1	97	0	0	3	398	0	0	6	122
χ^2 (*p*-value)	6.4(0.094)		3.5(0.315)		15.7(0.001)		11.2(0.011)		12.4(0.006)		27.3(<0.001)	
**Total**	36	259	41	296	34	245	27	268	21	152	159	243
	**HCV prevalence**											
**Blood type**												
O	0	0	0	0	1	19	0	0	0	0	1	4
A	3	89	1	30	3	89	1	41	1	30	9	57
B	2	49	2	48	3	73	1	34	1	25	9	47
AB	0	0	1	97	0	0	0	0	1	96	2	41
χ^2 (*p*-value)	5.1(0.164)		3.8(0.288)		3.0(0.391)		1.8(0.611)		3.9(0.268)		10.7(0.013)	
**Total**	5	36	4	29	7	50	2	20	3	22	21	32
	**HIV prevalence**											
**Blood type**												
O	1	19	0	0	0	0	1	25	0	0	2	8
A	1	30	0	0	4	119	2	83	2	60	9	57
B	4	97	1	24	0	0	2	69	0	0	7	36
AB	0	0	0	0	0	0	0	0	2	192	2	41
χ^2 (*p*-value)	4.1(0.246)		2.4(0.503)		12.5(0.006)		1.6(0.656)		13.4(0.004)		8.3(0.041)	
**Total**	6	43	1	7	4	29	5	50	4	29	20	31
	**Syphilis prevalence**											
**Blood type**												
O	2	37	6	111	7	130	2	50	3	56	20	78
A	0	0	7	212	3	89	4	165	4	120	18	114
B	6	146	6	145	7	170	8	275	6	149	33	171
AB	3	284	1	97	0	0	2	265	0	0	6	122
χ^2 (*p*-value)	11.8(0.008)		1.6(0.657)		2.3(0.504)		6.0(0.110)		3.3(0.342)		8.0(0.045)	
**Total**	11	79	20	144	17	122	16	159	13	94	77	118

Supplementary S3

Number of TTIs positive cases and TTIs prevalence by provinces with non-zero TTIs positive included in analysis.

Province	No. of donors	Number of TTIs positive					Pooled TTIs prevalence (pht)	Family income ($USD/month)
		HBV	HCV	Syphilis	HIV	Total		
Nakhon Si Thammarat	2,926	5	1	3	1	10	342	862
Krabi	576	3	0	0	0	3	521	846
Phangnga	360	1	0	0	0	1	278	682
Phuket	440	0	0	0	2	2	455	1015
Surat Thani	929	0	0	1	0	1	108	963
Songkla	48,913	122	15	56	14	207	423	630
Pattani	1,146	3	0	0	0	3	262	632
Phatthalung	2,794	5	0	6	2	13	465	640
Satun	1,132	1	4	3	1	9	795	668
Trang	1,649	1	1	1	0	3	182	731
Yala	939	2	0	0	0	2	213	518
Narathiwat	952	2	0	0	0	2	210	511
**Total**	62,756	145	21	70	20	256	-	-

Supplementary S4

Socio-demographic characteristics of blood donors who contributed to Songklanagarind Hospital from January 2018 to December 2022 comparing between pre-COVID-19 and during COVID-19 period.

Donor Characteristic	Pre-COVID19 (2018-2019)		COVID19 (2020-2022)		Δ% ($\%_{during}$- $\%_{pre}$)
	N	%	N	%	
**Gender**					
Male	12,774	46.0	18,163	48.1	2.1
Female	14,968	54.0	19,606	51.9	-2.1
**Age**					0
17-20	4,027	14.5	4,768	12.6	-1.9
21-30	9,823	35.4	12,342	32.7	-2.7
31-40	6,675	24.1	9,202	24.4	0.3
41-50	4,964	17.9	7,554	20.0	2.1
51-65	2,253	8.1	3,903	10.3	2.2
**Occupation**					0
Gov/State employee	4,950	17.8	8,406	22.3	4.5
Company employee	3,416	12.3	4,514	12.0	-0.3
Business owner	2,914	10.5	3,775	10.0	-0.5
Freelance	4,030	14.5	5,981	15.8	1.3
Farmer	630	2.3	942	2.5	0.2
High school student	1,442	5.2	1,658	4.4	-0.8
University student	7,672	27.7	8,784	23.3	-4.4
Cleric/Monk	127	0.5	143	0.4	-0.1
Other	2,561	9.2	3,566	9.4	0.2
**Blood type**					0
O	10,736	38.7	14,759	39.1	0.4
A	6,685	24.1	9,129	24.2	0.1
B	8,237	29.7	11,059	29.3	-0.4
AB	2,084	7.5	2,822	7.5	0
**Donor experience**					0
First-time donor	4,417	15.9	6,236	16.5	0.6
Repeat donor	23,325	84.1	31,533	83.5	-0.6
**Donation location**					0
At hospital	22,175	79.9	30,284	80.2	0.3
Mobile unit	5,567	20.1	7,485	19.8	-0.3
**Total**	**27,742**	**100.0**	**37,769**	**100.0**	-

Supplementary S5

At Blood Bank and Transfusion Medicine Unit, Department of Pathology, Songklanagarind Hospital, the guideline for blood donor was adapted from Thailand's national guidelines on blood donation of Thai Red Cross Society National Blood Centre.

The eligibility criteria for blood donation at the Blood Bank and Transfusion Medicine Unit.

Weight >45 kg (>53 kg for mobile donation unit)

Age 17-70 years old

Health condition

- Healthy and enough sleep of 6 hours
- No sign of fever, cold, cough, sore throat, runny nose, phlegm, or diarrhea
- No heart disease, liver disease, lung disease, blood disease, cancer, or tendency for easy bleeding
- Not infected with/being a carrier of hepatitis virus or TTIs
- Not having malaria in the past 3 years or having traveled to an area with high malaria transmission within the past year
- No history of major surgery, childbirth, or miscarriage within six months
- Women: not menstruating, pregnant, or breastfeeding
- Not tattooing, tattoos removing, ear piercing, or piercing of other body parts within 4 months
- No risky of sexual behavior
- No history of drug/substance abuse of any kind
